# Characterization of the Murine Myeloid Precursor Cell Line MuMac-E8

**DOI:** 10.1371/journal.pone.0113743

**Published:** 2014-12-29

**Authors:** Stephan Fricke, Cathleen Pfefferkorn, Doris Wolf, Sina Riemschneider, Janine Kohlschmidt, Nadja Hilger, Christiane Fueldner, Jens Knauer, Ulrich Sack, Frank Emmrich, Jörg Lehmann

**Affiliations:** 1 Fraunhofer Institute for Cell Therapy and Immunology (IZI), Leipzig, Germany; 2 Institute of Clinical Immunology, University of Leipzig, Leipzig, Germany; 3 Translational Centre for Regenerative Medicine, University of Leipzig, Leipzig, Germany; 4 Department of Surgery, Research Laboratories, University of Leipzig, Leipzig, Germany; Rutgers - New Jersey Medical School, United States of America

## Abstract

Starting point for the present work was the assumption that the cell line MuMac-E8 represents a murine cell population with stem cell properties. Preliminary studies already pointed to the expression of stem-cell associated markers and a self-regenerative potential of the cells. The cell line MuMac-E8 should be examined for their differential stage within stem cell hierarchy. MuMac-E8 cells were derived from a chimeric mouse model of arthritis. It could be shown that MuMac-E8 cells express mRNA of some genes associated with pluripotent stem cells (Nanog, Nucleostemin), of genes for hematopoietic markers (EPCR, Sca-1, CD11b, CD45), for the mesenchymal marker CD105 and of genes for the neural markers Pax-6 and Ezrin. In methylcellulose and May-Grünwald-Giemsa staining, hematopoietic colonies were obtained but the hematopoietic system of lethally irradiated mice could not be rescued. Osteogenic differentiation was not detectable. Thus, it became evident that MuMac-E8 represents not a stem cell line. However, MuMac-E8 cells expressed several myeloid surface markers (i.e. CD11b, F4/80, CD14, CD64), showed phagocytosis and is capable of producing nitric oxide. Thus, this cell line seems to be arrested an advanced stage of myeloid differentiation. Adherence data measured by impedance-based real-time cell analysis together with cell morphology data suggested that MuMac-E8 represents a new macrophage precursor cell line exhibiting weak adherence. This cell line is suitable as an *in-vitro* model for testing of macrophage functions. Moreover, it might be also useful for differentiation or reprogramming studies.

## Introduction

In recent years the investigation and characterization of new stem cell lines for improvement of cellular therapies came strongly into the focus of science. Because of their great potential they are a beacon of hope in areas of transplantation and regenerative medicine. However, the use of human embryonic stem cells for research purposes and its therapeutic application is both ethically and legally controversial. Accordingly, the establishment of suitable models allowing most realistic study of stem cells is necessary.

The cell line MuMac-E8 is a result of experiments in a chimeric mouse model of arthritis (human/murine SCID arthritis) [1], [2]. In that model, human synovial fibroblasts from patients with rheumatoid arthritis (RA) induced arthritis in SCID (severe combined immunodeficiency) mice. In following experiments, scientists tried to modulate this human/murine SCID arthritis by various cytokines. IL-4 is a potent suppressor of Th1-mediated mechanisms, which are still thought to play a role in various autoimmune diseases [3], [4]. For this purpose, IL-4-transfected murine fibroblasts (NIH-3T3BMG-Neo-IL-4) [5] were injected into the affected knee joint of mice three days after intraarticular application of human RA fibroblasts. Normal skin fibroblasts, NIH-3T3-IL-4 fibroblasts alone and NIH-3T3 fibroblasts transfected with empty BMG-Neo vector served as controls. Subsequently, the knee joint swelling was observed over 6 weeks. In this process the RA fibroblasts induced murine/human SCID arthritis worsened massively by injection of 3T3-IL-4 fibroblasts. There was a much stronger tumor-like swelling of the knees detectable compared to animals, which only RA synovial fibroblasts were injected. In all three control groups, however, there was observed only a transient moderate swelling of the treated knee joint (Lehmann, J. *et al.* unpublished data).

Pieces of the resulting tumor-like tissue were placed in culture in order to generate tumor cell lines for further characterisation. Outgrowing cells were cloned several times and stable cell clones were stored in liquid nitrogen. The cell line MuMac-E8 was one of these cell clones. In initial experiments, self-regenerative potential of MuMac-E8 cells could be confirmed using limiting dilution analysis. This raises the question whether the MuMac-E8 cell line revealed a stem-cell like phenotype and what differentiation potential they have or whether MuMac-E8 cells are suitable for research focusing on myeloid cells in various disease settings, especially in cancer. *In-vitro* culture systems allowing the production of myeloid cell subsets including myeloid suppressor cells that are found in the environment of cancers [6], [7] will give new insights in understanding the pathophysiology of tumor growth [6]–[8].

Here, we wanted to investigate the cell line MuMac-E8 in terms of their position within the hematopoietic lineage. In addition to immunophenotyping of MuMac-E8 cells by flow cytometry, the principal objective of this work was the establishment of quantitative real-time polymerase chain reaction (PCR) assays for gene expression analysis of stem-cell- and lineage-associated markers using the Universal Probe Library (UPL) method. The cells were locked in the G_0_ phase by synchronization using serum deprivation [9]–[11]. Then serum addition allowed the cells to re-enter to cell cycle.

After cell synchronization, the expression kinetics of several relevant genes was measured over 30 days. Using probe-based (UPL) quantitative real-time RT-PCR, changes in expression levels of selected pluripotency and differentiation markers could be identified. In addition, the differentiation potential of MuMac-E8 cells under different conditions was detected by appropriate *in-vitro* differentiation protocols and *in-vivo* experiments with lethally irradiated mice.

## Material and Methods

### Culture conditions of the cell line MuMac-E8

MuMac-E8 cells were cultured at a starting density of 5×10^5^ cells per well in 6-well plates (Nunc, Wiesbaden, Germany) or 75-cm^2^ cell culture flasks in RPMI 1640 Medium supplemented with 10% FCS, 100 U/ml penicillin, and 100 U/ml streptomycin (all from Biochrom, Berlin, Germany) at 37°C, 5% CO_2_ and 95% air humidity. Fresh culture medium was added twice a week and the cells were subcultured at 80% confluence by transferring non-adherent MuMac-E8 cells into a new cell culture flask (ratio 1∶3).

### Measurement of adherence and proliferation by real-time cell analysis

Real-time cell analysis using the xCELLigence RTCA system (xCELLigence RTCA SP instrument, ACEA, San Diego, CA, USA/Roche Diagnostics, Mannheim, Germany) represents a promising novel method for real-time analysis of adherence, proliferation, migration or cell death of adherent cells based on the application of electrical cell substrate impedance changes [12]. Electrical impedance is primarily determined by the ion environment both at the electrode-solution interface and in the bulk solution. The presence of cells affects the local ionic environment at the electrode solution interface. It varies according to cell size, cell morphology and strength of adhesion of the cells to the surface of the electrode and will result in change in the electrode impedance. The cells are seeded in the wells of an E-Plate 96 with interdigitated microelectrode arrays integrated in the bottom of each well. Subsequently, the E-Plate 96 is mounted on the SP Station of the xCELLigence RTCA system which is placed in a standard temperature-controlled CO_2_ incubator under humidity saturation. The RTCA Software preinstalled on the RTCA control unit allows automatic selection of wells for measurement and real-time data acquisition within preprogrammed time intervals. Cell status is represented by a dimensionless parameter termed Cell Index (CI) which is derived as the relative change in measured electrical impedance. This basically represents the changes in the impedance subtracted by background measurements from media alone.

As a prerequisite of an individual application for the xCELLigence RTCA system, the cells to be tested have to be optimized for their culture conditions within the E-Plate 96. In particular, the cell number has to be adjusted for optimum output. Therefore, MuMac-E8 were gently trypsinized, washed twice in pre-warmed culture medium (RPMI 1640, 10% FCS, 2 mM stable L-glutamine, 10 mM HEPES, 100 U/ml penicillin, 100 µg/ml streptomycin; Biochrom, Berlin, Germany), adjusted at different amounts of cell density (i.e. 1×10^2^, 3×10^2^, 1×10^3^, 3×10^3^, 1×10^4^, 3×10^4^, 1×10^5^ cells/well), and applied to an E-Plate 96. Medium alone was used for control. Then, cells were cultured over 48 h, and cell adhesion and proliferation were continuously monitored by the xCELLigence RTCA SP instrument.

### Synchronization of MuMac-E8 cells by serum deprivation

MuMac-E8 cells were adjusted at the G_0_ phase of the cell cycle through serum deprivation. Cells were cultured without FCS or with diminished supplementation of FCS (i.e. 0.1%, 0.5%, 2.5%, 5%) in comparison to normal culture conditions (i.e. 10% FCS) in order to identify the optimal conditions for synchronization of MuMac-E8 cells in terms of adjustment at the G_0_ phase and metabolic activity/vitality. Culture medium alone without cells was used to determine the background level. The starvation phase was continuously monitored by the xCELLigence RTCA system. In parallel, after 24 and 48 h the cell vitality or metabolic activity was analyzed using the WST-1 assay (Roche Diagnostics). In a successive experiment the cell cycle was re-entered after the optimum starvation time, as identified in the former experiment, by supplying 10% FCS. Restart of synchronized proliferation of MuMac-E8 cells was then analyzed utilizing the RTCA SP Instrument for another 72 h.

### RNA isolation and real-time RT-PCR for selected marker genes

**Figure 1:**
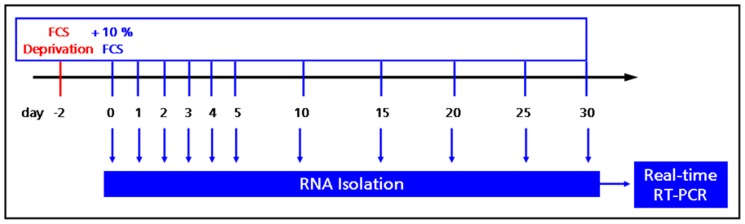
Experimental strategy for gene expression analyses. MuMac-E8 cells harvested from bulk culture were synchronized by serum deprivation for 48 h. The cell cycle was re-entered by supplementation of the culture medium with 10% FCS. At indicated time points total RNA was isolated for subsequent gene expression analysis by real-time RT-PCR.

MuMac-E8 cells derived from bulk culture were washed twice, adjusted in fresh culture medium (RPMI1640/10% FCS, Biochrom), plated into 6-well plates (Nunc; 5×10^5^ cells/well) and were allowed to adhere over night at 37°C, 5% CO_2_ and 96% humidity. Then, culture medium was removed, cells were washed twice by carefully rinsing with 37°C-warm PBS and 3 ml serum-free medium were added per well. After 48 h the serum-free medium was replaced by complete culture medium. At different time points total RNA as extracted and transcribed *in vitro* in the corresponding cDNA (Fig. 1). The quantification of the RNA was determined by UV spectroscopy at 260/280 nm (NanoDrop ND-1000, PEQLAB, Erlangen, Germany). The cDNA synthesis was performed using the Transcriptor First Strand cDNA Synthesis Kit according to manufacturer's instructions. The *in-vitro* reverse transcription (RT) was carried out in a conventional thermocycler (TProfessional, Biometra, Göttingen, Germany) initially at 25°C for 10 min, followed by a 30-minute reaction period at 55°C and transcription for 15 min at 85°C. The reaction was stopped by cooling on ice. To quantitatively determine the gene expression of selected pluripotency and differentiation markers, quantitative real-time PCR assays were established using UPL oligonucleotide probes (Roche Diagnostics). The probe and primer design was done with the web-based ProbeFinder software. Selected primers and corresponding UPL probes are listed in table 1. For real-time PCR, the LightCycler 480 Probes Master Kit (Roche Diagnostics) was used in combination with UPL probes and corresponding primer pairs. The PCR was carried out on the LightCycler 480 instrument (Roche Diagnostics) in detection format Mono Color Hydrolysis Probe. For analysis of relative gene expression of MuMac-E8 cells, aminolevulinic acid synthase 1 (ALAS1) and porphobilinogen-deaminase (PBGD) were used as housekeeping genes. Amplification was performed using the following conditions: 10 min activation and denaturation step at 95°C, followed by 50 repetitive cycles of denaturation at 95°C for 10 s, annealing at primer-specific annealing temperature for 30 s and polymerization at 72°C for 1 s. The analysis of relative gene expression was done by using LightCycler 480 Relative Quantification Software.

**Table 1:** Sequences of primers and corresponding UPL probes for selected marker genes.

Marker gene	Forward primer	Reverse primer	UPL probe
AFP	catgctgcaaagctgacaa	ctttgcaatggatgctctctt	63
ALAS1	ccctccagccaatgagaa	gtgccatctgggactcgt	40
Beta-III-Tubulin	gcgcatcagcgtatactacaa	ttccaagtccaccagaatgg	104
BMP-4	gaggagtttccatcacgaaga	gctctgccgaggagatca	89
Brachyury	cagcccacctactggctcta	gagcctggggtgatggta	100
CD117	gatctgctctgcgtcctgtt	cttgcagatggctgagacg	15
CD11b	aaggatgctggggaggtc	gtcataagtgacagtgctctggat	16
CD14	aaagaaactgaagcctttctcg	agcaacaagccaagcacac	25
CD34	atgaaccgtcgcagttgg	ccgtgtaataagggtcttcacc	3
CD38	aagatgttcaccctggagga	ctccaatgtgggcaagagac	99
CD45	agttagtgaatggagaccaggaa	tccataagtctgctttccttcg	40
CD80	ttcgtctttcacaagtgtcttca	tgccagtagattcggtcttca	31
CD86	gaagccgaatcagcctagc	cagcgttactatcccgctct	47
CD90	tcccatgagctccaataaaag	gaggagggagagggaaagc	19
Endoglin (CD105)	aaatcccgttgcacttgg	actcttggctgtccttggaa	66
EPCR	agcgcaaggagaacgtgt	gggttcagagccctcctc	34
Ezrin	agccgaatagccgaggac	gtcacccggacgttgatt	29
GATA-1	gaatcctctgcatcaacaagc	gggcaagggttctgaggt	67
GFAP_1	acagactttctccaacctccag	ccttctgacacggatttggt	95
GFAP_2	tcgagatcgccacctacag	gtctgtacaggaatggtgatgc	110
Goosecoid	gagacgaagtacccagacgtg	gcggttcttaaaccagacctc	1
Nanog	ttcttgcttacaagggtctgc	agaggaagggcgaggaga	95
Nestin	ctgcaggccactgaaaagtt	tctgactctgtagaccctgcttc	4
NST	accaagccagacagaagacc	ctacaggggccacatctgag	100
Oct-4	gttggagaaggtggaaccaa	ctccttctgcagggctttc	29
Osteocalcin	gccctgagtctgacaaaggta	ggtgatggccaagactaagg	79
Pax-6_1	acccggcagaagatcgtag	tttgcatctgcatgggtct	68
Pax-6_2	gcttggtggtgtctttgtca	tgcatctgcatgggtctg	63
PBGD	tccctgaaggatgtgcctac	acaagggttttcccgtttg	92
PDGFRα	ggttgttgatctgcagtgga	cctccagcatggtgatacct	25
Sca-1_1	tgaagacattttggaattaatgga	tcaccatggcttagaatcaca	26
Sca-1_2	gaggctgacttcctgtatgctt	cacgacccgtcctttgaa	91
Sca-1_3	cccctaccctgatggagtct	tgttctttactttccttgtttgagaa	107
Sox-2	tccaaaaactaatcacaacaatcg	gaagtgcaattgggatgaaaa	80
Wnt5a	tgaagcaggccgtaggac	agccagcacgtcttgagg	16

### Colony-forming cell assay

The hematopoietic potential was investigated by factor-induced differentiation-experiments on methylcellulose medium (MethoCult M3434 methylcellulose medium, StemCell Technologies, Vancouver, Canada), so-called colony-forming cell assay (CFC assay). 2×10^5^ cells/ml medium were suspended. Then, 0.3 ml of this cell suspension was added to 3 ml MethoCult, mixed and added on a 35-mm HydroCell culture dish (Nunc; characterized by very low cell attachment). Some colonies were prepared with May-Grünwald-Giemsa staining. The cells were cultured for 12 days in an incubator and examined periodically using an inverted microscope (Axiovert 40, Carl Zeiss MicroImaging, Jena, Germany). After 12 days final microscopic images were made.

### Osteogenic and chondrogenic differentiation

The cells were cultured for 14 days in osteogenic differentiation medium. As proof of chondrogenesis collagen staining was done. The cells were grown in increasing amounts of cell density (1×10^3^, 1×10^4^, 1×10^5^ cells/culture dish) seeded in 10-cm culture dishes (Nunc) that contained 8 ml of osteogenic differentiation medium. After 14 days of culture at 37°C, 5% CO_2_ and 95% air humidity, the cells were fixed with ice-cold 70% ethanol for 10 min at 4°C. Subsequently, several stainings were carried out for the detection of differentiation (visualization of alkaline phosphatase, calcium staining, collagen staining, methylene blue staining).

### Cell transplantation

Corresponding numbers of cells (1×10^6^, 2×10^6^ cells) were added under sterile conditions in 150 µl 0.9% sterile, physiological saline and aliquoted into sterile 1.5-ml Eppendorf tubes. Until transplantation, the cells were stored at 4°C. The grafts were subsequently injected intravenously into the lateral tail vein of lethally irradiated (8 Gy) recipient mice. The observation of the results of transplantation was carried out by daily weight and survival control of these animals. The blood count was checked by weekly retro-orbital blood sample over 50 days. The leukocyte count was determined using the analyzer Scil Vet abc (Scil animal care company, Viernheim, Germany).

### Animals and irradiation protocol

The triple-transgenic recipient mice (TTG) are murine CD4^-/-^ mice expressing the human CD4 and HLA-DR3 molecules on a stable C57Bl/6 background [13]–[17]. These mice were bred and maintained under standardized conditions at the Medical Experimental Center of the University of Leipzig. The CD4 transgene includes its own promoter ligated to a murine CD4 enhancer element thus leading to T-cell subset-specific expression. CD8^+^ cells are not affected in TTG mice and express the HLA-DR3 molecule in addition to the murine MHC II complex [13]–[17]. Furthermore, the TTG mice have a complete functional murine immune system which is modified with regard to CD4 and HLA-DR [9]–[13]. The irradiation of TTG mice was previously described [13]. In brief, 4 animals were irradiated with 8 Gy in parallel with a lethal irradiation dose determined before with 0.4 Gy/g in a Plexiglas container under the X-Ray apparatus (D3225, Orthovoltage, Gulmay Medical, Camberley, UK). After transplantation, recipient TTG mice were kept under antibiotic therapy (Baytril 2.5% ad us. vet., Bayer Animal Health, Leverkusen, Germany) for 14 days [13]–[17].

### Ethics statement

All mice were housed, treated and handled under permission and in accordance with the guidelines of the Animal Care Committee of the University of Leipzig and the Regional Board of Animal Care for the district of Leipzig (animal experiment registration numbers 24/06, 28/08, and 55/11). Especially in the permission for 28/08 “Tolerance through regulatory immune cells”, the Animal Care and Use Committee specifically approved this study to investigate the behavior of immune cells after hematopoietic stem cell transplantation with regard to engraftment and immunological impact.

### Fluorescence staining of F-actin with Phalloidin-Alexa

By means of Phalloidin, a bicyclic heptapeptide which represents one of the toxins of the “death cap” mushroom (*Amanita phalloides*), intracellular F-actin filaments can efficiently be stained. Therefore, adherent MuMac-E8 cells cultured in chamber slides (Nunc) for 2 days (2×10^4^ cells/well) were fluorescence-stained with Phalloidin-Alexa (Sigma, Taufkirchen, Germany). Prior to staining the adherent cells were washed twice with 500 µl cold PBS, fixed with 100 µl ice-cold 4% paraformaldehyde for 10 min and successively perforated by washing three times with 500 µl 0.1% saponin (Sigma) in PBS. Then, Phalloidin-Alexa was added and incubated over night at 4°C on a shaker. Finally, the top frame of the chamber slide was removed, the slides washed twice in PBS and once in deionized water and then embedded in Fluorescent Mounting Medium (DAKO, Hamburg, Germany). Slide assessment and preparation of microphotographs were done by means of a confocal laser-scanning microscope (LSM 510 Meta, Zeiss, Oberkochen, Germany).

### Immunophenotyping by flow cytometry

The phenotype of MuMac-E8 cells was characterized by flow cytometry. Cells were harvested from the culture flask, washed twice in PBS and subsequently 1×10^6^ cells were stained simultaneously with mAbs recognizing CD11b (clone M1/70), F4/80 (clone BM8), CD64 (clone X54-5/7.1; all from BioLegend, London, UK) and CD14 (clone Sa2-8) (eBioscience, Frankfurt, Germany) or with the respective isotype controls Rat IgG2a (clone RTK2758; BioLegend) for 30 min at 4°C in the dark, washed three times in cold PBS, and measured using a FC500 flow cytometer (Beckman Coulter, Krefeld, Germany). Results were calculated using the CXP analysis software (Beckman Coulter).

### Measurement of phagocytosis by imaging flow cytometry

For measurement of phagocytic potential, MuMac-E8 cells were harvested and 1×10^6^ cells were incubated for 2 h with 2×10^7^ FITC-labeled heat-killed *Salmonella* Enteritidis bacteria. Afterwards, cells were washed 4 times with HBSS and the uptake of bacteria by MuMac-E8 cells was assessed by imaging flow cytometry using the novel imaging flow cytometer Amnis FlowSight (Merck Millipore). This novel technology allows not only the quantitative flow cytometric determination of the number of fluorescence-positive cells but also the qualitative confirmation of incorporated fluorescence-labeled bacteria inside of the analyzed cells by performing images of the cells during analysis.

### NO assay

The amount of nitrite and nitrate in cell culture supernatants is an indicator for the production of short-living NO radicals in the cells [18]. The nitrite and nitrate concentration in supernatants of MuMac-E8 cells was measured using the Griess reaction as described elsewhere [19]. Briefly, 50 µl cell culture supernatant were transferred into a 96-well microtiter plate. As a reference standard 20 mM NaNO_2_ (stock solution in ultrapure water) was firstly 1∶20 diluted in culture medium followed by log_2_-titration down to 0.1 µM in order to generate a calibration curve. Then, equal volumes of Griess reagents A (0.1% w/v N-(1-Naphtyl)-ethylendiamin-dihydrochlorid in absolute Ethanol) and B (1% v/v Sulfanilamin in 5% v/v H_3_PO_4_) were mixed and 100 µl of the ready-prepared Griess reagent were added to the wells containing culture supernatants and to the wells for the calibration curve as well. After 10 min of incubation at room temperature in the dark the optical density was measured at a wavelength of 550 nm using a multimode microplate reader (Safire2, Tecan Group, Crailsheim, Germany). Results were calculated using the Magellan software (Tecan Group).

The specificity of iNOS-dependent NO production was shown by addition of the iNOS inhibitor AMT-HCl (Alexis, Grünberg, Germany; final concentration 50 µM, 1% v/v DMSO).

### Statistical analysis

The data are given as means ± standard deviation. Statistical analysis and diagrams were generated using SigmaPlot 10.0/SigmaStat 3.5 software (SYSTAT, Erkrath, Germany) as well as the RTCA Software. Kaplan-Meier survival curves were analyzed using the Log-rank test and pair wise multiple comparisons of means (Holm-Sidak method).

## Results

### Adherence and proliferation profile of MuMac-E8 cells

**Figure 2:**
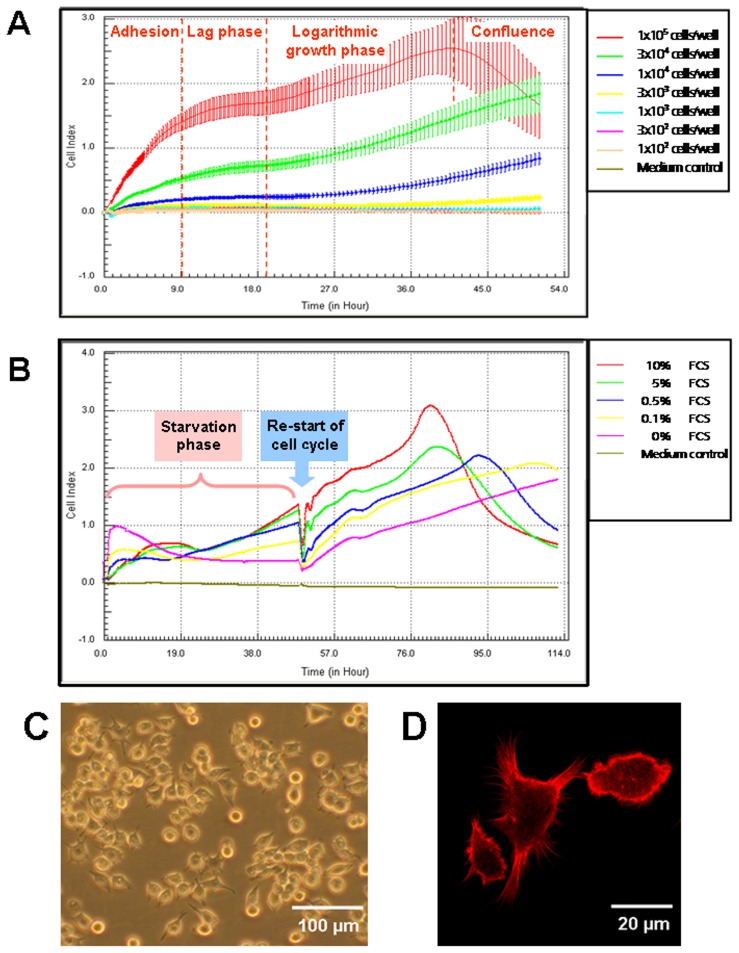
Optimum cell culture conditions and morphology of cultured MuMac-E8 cells. By means of real-time cell analysis using the xCELLigence system the optimum cell density (n = 12 per value of cell density, mean ± SD; A) and the optimum serum supplementation for cell synchronization through serum deprivation (B) were determined. Typical morphology of MuMac-E8 cells in culture was shown by inverse phase contrast microscopy (Axiovert, Zeiss). The majority of the cells were found to grow adherent until confluence (C). Visualisation of cell spreading after fluorescence staining of F-actin with Phalloidin-Alexa was performed by confocal laser-scanning microscopy (LSM 510 Meta, Zeiss) (D). Figure was reprint from Biochemica, 4, 14–16 [12] with permission from the journal.

The adherence behavior of MuMac-E8 cells was studied by real-time cell analysis using the xCELLigence RTCA system (ACEA/Roche Diagnostics). Using this novel methodology, it was shown that the adherence phase took approximately 12 h, followed by a lag phase with no further change of the cell index (relative dimension of the impedance signal) up to 18 h, before the cells start to proliferate indicated by a further linear increase of the cell index (Fig. 2A). For RTCA experiments between 1×10^4^ and 1×10^5^ cells/well revealed to be applicable. However, as shown in figure 2A the confluence stage was reached earlier (after ≈40 h) if a starting cell density of 1×10^5^ cells/well was used, indicated by decreasing CI values likely caused by loosing adherence rather than dying of the cells.

MuMac-E8 cells express a heteromorphic phenotype in culture. Non-adherent and adherent cells could be observed side by side. While the non-adherent cells seemed to represent cells in the mitotic phase, adherent cells seemed to represent either a post-mitotic phenotype in the G_0_ phase or the inter-phase phenotype (G_1_, S- or G_2_-phase) of the cell cycle, as concluded from observations in several time-lapse videos of MuMac-E8 cells cultured under conventional conditions (Video S1). By means of fluorescence-staining of post-mitotic adherent cells with Phalloidin-Alexa F-actin filaments in cytoplasm appendices could be made visible, underlying the macrophage morphology of differentiated MuMac-E8 cells (Fig. 2C+D).

### Synchronization of MuMac-E8 cells

As shown in Figure 2B, only starvation of MuMac-E8 cells without FCS (0% FCS) prevented proliferation over a period of 48 h. In contrast, diminished serum supplementation even at very low levels (i.e. 0.1%, 0.5%) led to proliferation of MuMac-E8 cells indicated by increasing CI values starting 30 h after the cells were seeded into the E-Plate 96. However, a serum concentration of 2.5% induced a proliferation curve reaching nearly the dynamics of 10% FCS content (not shown). Finally, no differences could be found between the proliferation curves of 5% and 10% FCS suggesting saturation of essential growth factors at serum contents of more than 5% FCS. Interestingly, the different FCS contents in the culture medium caused remarkable differences during the adhesion phase. Without FCS, MuMac-E8 cells attached more efficiently to the E-Plate 96 surface and also 0.1% and 0.5% FCS allowed a faster and more efficient adherence than supplementation of 2.5% (not shown), 5%, and 10% FCS. This phenomenon might be explained by blocking molecules responsible for cell adherence by soluble serum factors. To confirm that the cells stayed alive over starvation periods of 24 h and 48 h, the cell viability/metabolic activity was proved by using the WST-1 assay. The delivered WST-1 signal indicated similar cell viability/metabolic activity in MuMac-E8 cultures containing 0%, 0.1%, and 0.5% FCS. Although 2.5-10% FCS caused higher WST-1 signals than lower FCS concentrations, the cells cultured for 48 h without FCS were found to be alive, too (data not shown). Summarizing these data, MuMac-E8 cell can be starved for a period of 48 h with complete deprivation of serum. These conditions are appropriate for quiescence of the cells to the G_0_ phase.

To verify that the cells are capable of re-entering the cell cycle after 48 h of total serum deprivation by substitution of complete culture medium, containing 10% FCS, proliferation curves were analyzed with the xCELLigence RTCA system. As shown in Figure 2B, the G_1_/S phase of the cell cycle could be re-started in all samples. However, the level of CI values correlated strictly with the FCS concentration during the starvation phase. Nevertheless, it could clearly be shown that even if MuMac-E8 cells were cultured without FCS they started to proliferate following supply of 10% FCS.

### Gene expression profile of MuMac-E8 analyzed by real-time RT-PCR

**Figure 3:**
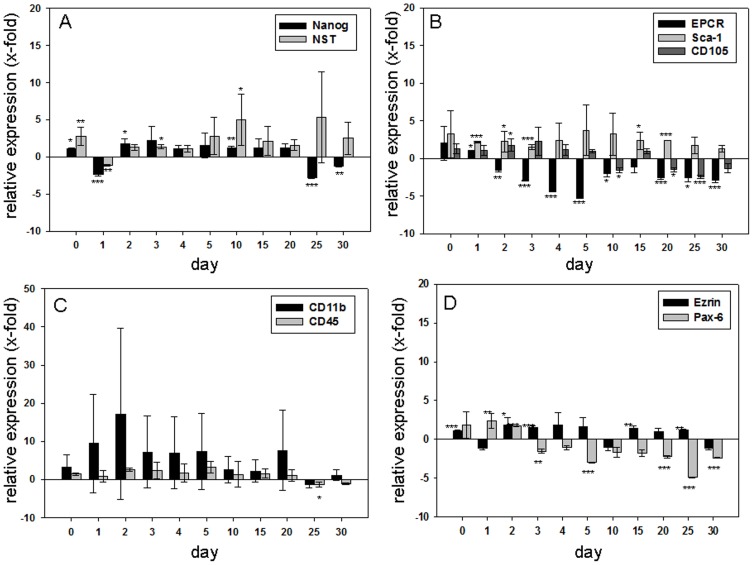
Relative mRNA expression of several pluripotency and differentiation markers. (A) Relative expression of the marker of pluripotency Nanog and Nucleostemin (NST) determined by quantitative real-time RT-PCR (n = 6 per gene). Shown is the resultant of two independent experiments, mean ± SD. The expressions are shown in relation to control cells (prior to synchronization). Negative values stand for a reduced expression, positive values for an increased expression of each gene compared to control cells. Nanog was hardly regulated. There were only small increases or decreases in expression. Nucleostemin (NST) showed on day 10 and 25 a 4-fold increase in expression. On all other days of culture variations were found around the control value. (B) Relative expression of the hematopoietic markers EPCR, Sca-1 and the mesenchymal marker CD105 determined by quantitative real-time RT-PCR (n = 6 per gene). Shown is the resultant of two independent experiments, mean ± SD. The expressions are shown in relation to control cells (prior to synchronization). Negative values stand for a reduced expression, positive values for an increased expression of each gene compared to control cells. EPCR showed in the first days after synchronization a significant decrease in expression and approached back to the expression level of control cells. In Sca-1 a slightly increased expression was observed, while it approached to the control value 30 days after treatment. CD105 expression rate decreased slightly within 30 days. (C) Relative expression of the hematopoietic markers CD11b and CD45 determined by quantitative real-time RT-PCR (n = 6 per gene). Shown is the resultant of two independent experiments, mean ± SD. The expressions are shown in relation to control cells (prior to synchronization). Negative values stand for a reduced expression, positive values for an increased expression of each gene compared to control cells. CD11b expression showed a clear increase up to day 2. Until day 30 the expression levels of CD11b approached to the control value. For CD45, only minor fluctuations were determined around the control value. (D) Relative expression of the neuronal markers Ezrin and Pax-6 determined by quantitative real-time RT-PCR (n = 6 per gene). Shown is the resultant of two independent experiments, mean ± SD. The expressions are shown in relation to control cells (prior to synchronization). Negative values stand for a reduced expression, positive values for an increased expression of each gene compared to control cells. Ezrin showed very moderate expression increases and reductions, which followed no clear pattern. Pax-6 expression was found to be decreased up to 5-fold of the control level between days 3 and 30.

We determined the gene expression of pluripotency, hematopoietic, mesenchymal, and neuronal markers to characterize the MuMac-E8 cell line. The mRNA expression profile of MuMac-E8 cells was analyzed following *in-vitro* RT into cDNA by quantitative real-time PCR at different time points over 30 days. The relative expression of investigated markers was studied in relation to their expression in cells before synchronization (Fig. 3A–D). Cell synchronisation should arrest all MuMac-E8 cells in the experiment at the same stage of the cell cycle. The subsequent addition of serum should induce a spontaneous differentiation of the cells indicated by the expression of linage markers or an increase in the expression of stem cell markers. Indeed, an increase of pluripotency markers of the cells could be achieved. The evaluation of data was performed by the LightCycler 480 Relative Quantification Software. The individual samples were normalized using the reference genes ALAS and PBGD.

The pluripotency markers Oct-4 and Sox-2, the mesodermal markers BMP-4, Wnt5a, Brachyury and Goosecoid, the hematopoietic markers CD90.1, CD34, CD38, CD117, GATA-1, the fibroblast marker CD140a and the endodermal marker AFP were not detectable at the mRNA level. However, in MuMac-E8 cells, we were able to detect mRNA expression of the pluripotency markers Nanog and Nucleostemin (NST) (Fig. 3A), the hematopoietic markers EPCR, Sca-1, CD11b, and CD45 (Fig. 3B+3C), the mesenchymal marker CD105 (Fig. 3B) and the neuronal markers Ezrin and Pax-6 (Fig. 3D) by real-time RT-PCR. The expression of both pluripotency markers, Nanog and NST, was nearly about their expression in control cells (cells at day 0). Nanog showed a slight reduction of its expression up to day 30. The NST expression pointed out a slight increase by factor 2 at the start of the experiment. A phase followed in which expression level fluctuates around the control value. Between day 4 and day 10, a slight increase in expression of NST was observed up to 4.5 times, which came down again to the value of control cells on day 20. Within the first 5 days after culture synchronization, EPCR showed a significant decrease in expression. On day 5, the expression of EPCR was about 5 times reduced. From day 10, the expression approximated again to the level of expression in control cells. The expression of Sca-1 was increased in the first days of culture and approached from day 5 until the end of the tests steadily to the control value. CD105 showed a decrease in expression levels during the cultivation until day 30. A strong increase in the expression of CD11b could be detected up to 15 times on day 2 after cell synchronisation. Subsequently, its expression was reduced and reached the level of the control cells again until day 30. CD45 showed only small fluctuations around the control value.

The expression level of Ezrin was nearly unchanged up to day 30 in comparison to control cells on day 0, however weakly decreased expression was observed on days 1, 10 and 30. The level of Pax-6 mRNA expression was found to be decreased up to 5-fold of the control level between days 3 and 30 after restart of the cell cycle (Fig. 3D).

Besides the above described marker genes, mRNA expression of the neuronal marker genes nestin, beta-III-tubulin, GFAP and the osteoblast marker osteocalcin was detected. However, these genes were only very weakly and not continuously expressed over the period of 30 days of culture (data not shown). An overview of the mRNA expression profile of all tested genes in MuMac-E8 cells is given in table 2.

**Table 2:** Overview of the expression of investigated marker genes.

Investigated marker category	Expression detectable	Expression not detectable
marker of pluripotency	Nanog, Nucleostemin, Sca-1, EPCR, CD45, CD11b	Oct-4, Sox-2
hematopoietic marker	CD80, CD86, CD14	CD38, CD34, CD117, CD90, GATA-1
mesenchymal marker	CD105, Ezrin, Pax-6, Nestin, GFAP	CD90
neuronal marker	Beta-III-Tubulin	
marker of osteocytic differentiation	Osteocalcin	
endodermal marker		AFP
fibroblast marker		CD140a (PDGFRα)
mesodermal marker		BMP-4, Wnt5a, Brachyury, Goosecoid

### Potential of MuMac-E8 cells to form hematopoietic colonies

**Figure 4:**
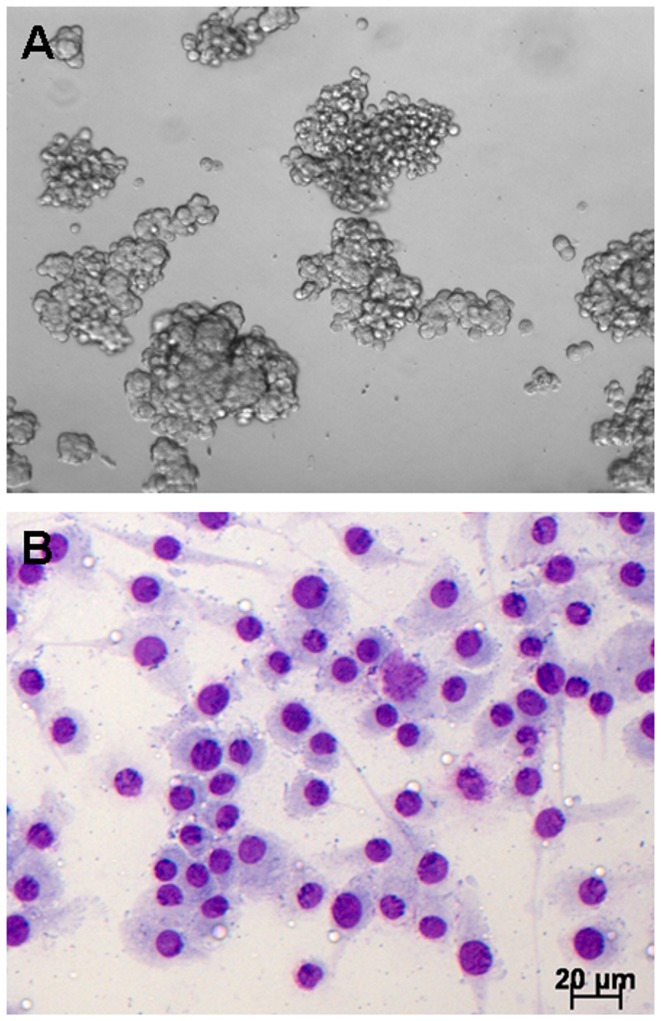
CFC assay after 12 days of culture. (A) MuMac-E8 cells were cultured for 12 days in methylcellulose-based semi-solid medium. The initial cell number was 2×10^5^ cells (magnification 100-fold). Cells revealed a distinct colony formation. The colonies consist of numerous cells of predominantly round shape. By comparison with example images from the manufacturer's instructions MuMac-E8 colonies were identified as CFU-M. (B) May-Grünwald-Giemsa stained MuMac-E8 cells harvested from bulk culture and allowed to adhere in chamber slides.

In order to identify hematopoietic progenitor cells *in vitro*, the methylcellulose-based CFC assay was used (Fig. 4A). This system allows the formation of myeloid and erythroid colonies from hematopoietic progenitors. Colonies in this assay showed a distinct colony formation and had a round shape. Comparison with exemplarily shown CFU-M colonies from manufacturer's instruction revealed a similar morphology, suggesting that MuMac-E8 cells belong to the monocyte/macrophage lineage. May-Grünwald-Giemsa staining of MuMac-E8 cells allowed to adhere in chamber slides revealed a typical monocyte/macrophage morphology (Fig. 4B).

### MuMac-E8 cells did not reveal osteogenic differentiation potential

**Figure 5:**
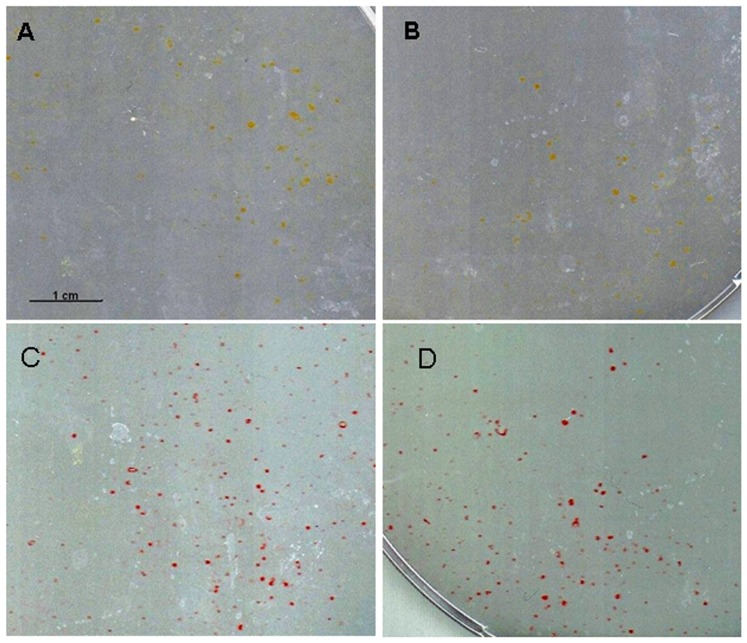
Alkaline phosphatase and calcium staining of MuMac-E8 cells. Used cell number was 1×10^4^. (A) The cultivation was carried out in osteogenic differentiation medium and in (B) normal medium. Isolated yellow-colored cells are visible in both cases. (C) The cultivation was carried out in osteogenic differentiation medium and in (D) normal medium. Isolated red-colored cells are visible in both cases.

**Figure 6:**
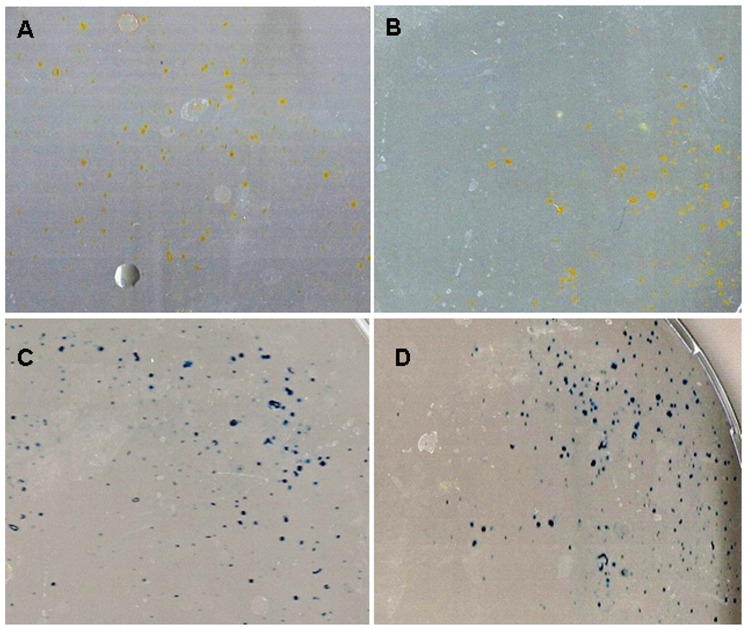
Collagen and methylene blue staining of MuMac-E8 cells. Used cell number was 1×10^4^. (A) The cultivation was carried out in osteogenic differentiation medium and in (B) normal medium. Isolated yellow-colored cells are visible in both cases. (C) The cultivation was carried out in osteogenic differentiation medium and in (D) normal medium. Isolated blue-colored cells are visible in both cases. There was no colony formation.

To study whether the MuMac-E8 cells have osteogenic potential the cells were cultured for 14 days in dexamethasone and ascorbic acid containing osteogenic differentiation medium (Fig. 5–6). Afterwards, different cytochemical staining was performed. By staining of alkaline phosphatase neither in the osteogenic differentiation medium (Fig. 5A) nor in normal medium (Fig. 5B) a red coloration of the cells as a sign for osteogenic differentiation was visible. The cells were only slightly yellow-colored. Calcium staining showed a weak red color of the cells in both the osteogenic differentiation medium (Fig. 5C) and in normal medium (Fig. 5D). With collagen staining (Fig. 6 A, B) in both media again only yellow-colored cells were visible. Staining with methylene blue (Fig. 6 C, D) revealed no evidence of colony formation. Only isolated cells were detectable.

### Failure of *in-vivo* reconstruction of the hematopoietic system by MuMac-E8 cells

**Figure 7:**
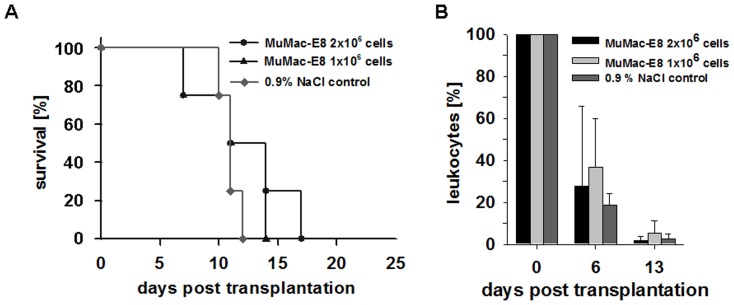
Kaplan-Meier survival curve and number of leukocytes in peripheral blood after transplantation of MuMac-E8 cells into mice. (A) Survival of lethally irradiated mice after administration of different cell numbers of MuMac-E8. Animals that have received a transplantation dose of 2×10^6^ cells survived up to 17 days. Following the administration of 1×10^6^ the survival decreased down to 14 days. The control animals died within 12 days. (n = 4 per group) (B) Level of leukocytes in peripheral blood determined by analysis of blood, n = 4 per group, mean ± SD. Leukocytes decreased within 13 days post transplantation in all groups (no engraftment).

Another possibility for the characterization of stem cells is represented in functional assays *in vivo* (reviewed in description, Ploemacher *et al.* [20]. Transplantation of MuMac-E8 cells into lethally irradiated mice should reveal whether this cell population is capable of reconstituting the hematopoietic system. After engraftment of 2×10^6^ MuMac-E8 cells animals survived up to 17 days, after administration of 1×10^6^ MuMac-E8 cells, up to 14 days. Control animals died within 12 days after irradiation (Fig. 7A). Blood samples were analyzed weekly to detect the number of leukocytes in the blood of transplanted mice. There was a significant decrease of the number of leukocytes in the blood of MuMac-E8 transplanted mice. Six days after transplantation, the number of leukocytes was found to be decreased to about 25–30%. On day 13 virtually no leukocytes were detectable. A similar reduction of the leukocyte number was observed in the control group (Fig. 7B).

### Immunophenotype of MuMac-E8 cells

**Figure 8:**
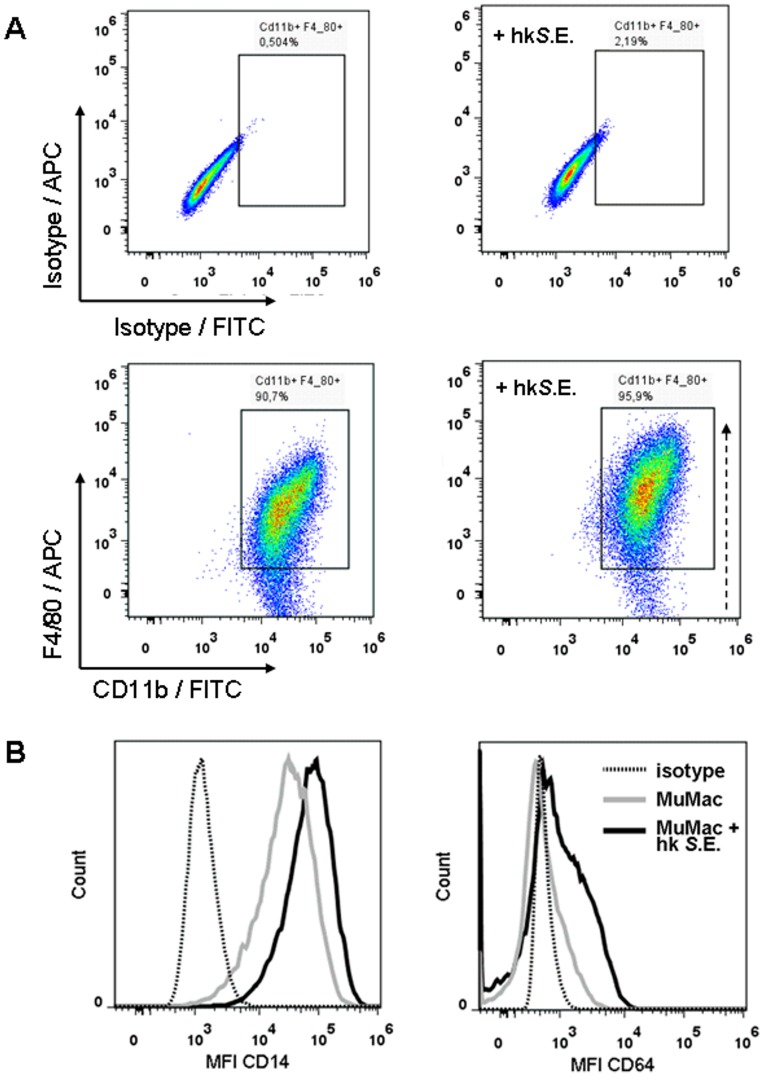
Immunophenotyping of MuMac-E8 cells. MuMac-E8 cells were stained with fluorochrome-labeled monoclonal antibodies recognizing the surface marker proteins CD11b, F4/80 (A), CD14, and CD64 (B) in order to identify the functional phenotype of this cell line. It could be shown that MuMac-E8 cells in general express CD11b and that the majority of cultured MuMac-E8 cells express also F4/80 (91%). The number of CD11b^+^/F4/80^+^ cells increased following co-incubation with heat-killed *S*.E. (96%; A, right-hand panel). CD11b^+^/F4/80^+^ MuMac-E8 cells revealed high or moderate surface expression of CD14 or CD64, respectively. Moreover, the expression of both CD14 and CD64 on CD11b^+^/F4/80^+^ MuMac-E8 cells was additionally inducible by stimulation with *Salmonella* antigen (B).

MuMac-E8 cells were stained with fluorochrome-labeled monoclonal antibodies recognizing the surface marker proteins CD11b, F4/80, CD14, and CD64 in order to identify the functional phenotype of this cell line. The cells revealed a typical macrophage staining pattern with high mean fluorescence intensity values of the markers CD11b and F4/80 (Fig. 8A left). As a functional characteristic it could be shown that the expression rate of F4/80 (MFI) and also the relative number of F4/80^+^ cells increased after stimulation with heat-killed salmonellae (Fig. 8A right). Moreover, MuMac-E8 cells revealed high surface expression of CD14 and moderate expression of CD64 (Fig. 8B). Both of these molecules were found to be up-regulated after stimulation with heat-killed salmonellae (Fig. 8B).

### Phagocytosis of heat-killed salmonellae by MuMac-E8 cells

**Figure 9:**
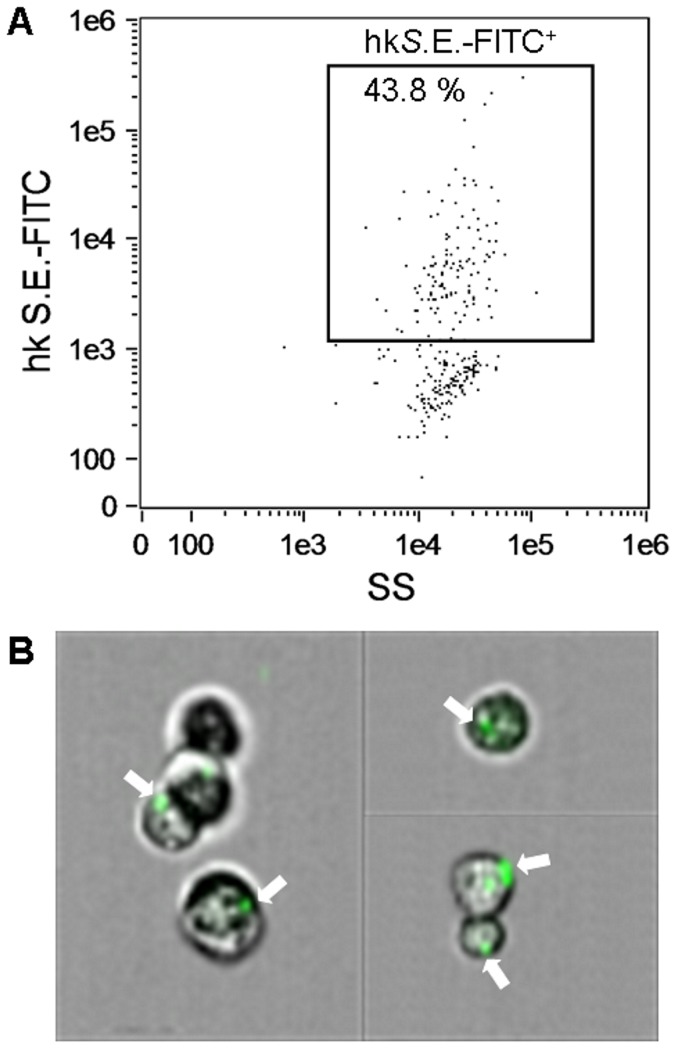
Phagocytic activity of MuMac-E8 cells. For measurement of phagocytic potential, MuMac-E8 cells were harvested and 1×10^6^ cells were incubated for 2 h with 2×10^7^ FITC-labeled heat-killed salmonellae. Afterwards cells were washed 4 times with HBSS and the uptake of bacteria was assessed by imaging flow cytometry (Amnis FlowSight system, Merck Millipore). In this representative experiment 44% of the cells revealed a positive signal (A). By parallel imaging it could be confirmed that the positive fluorescence signal coincided with phagocytosis of salmonellae. White arrows show cells with internalized FITC-labeled bacteria (B).

Phagocytosis represents an important feature of macrophages. Therefore, MuMac-E8 cells were incubated with FITC-labeled heat-killed salmonellae and quantitatively analyzed by imaging flow cytometry. In a representative experiment 44% of MuMac-E8 cells showed a positive signal indicating phagocytic activity (Fig. 9A). Images in figure 9B demonstrate visually that MuMac-E8 cells could efficiently engulf fluorescence-labeled bacteria.

### NO production of MuMac-E8 cells

In order to test the microbicidal capacity of MuMac-E8 cells in response to different bacterial stimuli, we have co-incubated the cells with either lipopolysaccharide (LPS, 1 µg/ml), heat-killed or viable salmonellae in several doses. Neither LPS nor heat-killed salmonellae were capable of inducing NO production in MuMac-E8 cells. In contrast, with the optimal concentration of viable salmonellae (i.e. 10^8^ CFU/ml) significant levels of NO could be induced. NO production was extensively abolished after co-incubation with the iNOS-specific inhibitor AMT-HCl indicating the iNOS dependency of NO synthesis (Tab. 3).

**Table 3:** NO production of MuMac-E8 cells. Nitrite concentration in supernatants of MuMac-E8 cells after 20 hrs of stimulation. The iNOS dependency of NO production was indicated by blocking the result under co-incubation with the specific iNOS inhibitor AMT-HCl. (CE – cell equivalents).

Stimulus	Concentration	NO_2_ [µM/ml]	NO [µM/ml] + 50 µM AMT-HCl
Medium control	-	0.3	0.5
DMSO control	1% v/v	0.0	0.1
LPS *E.coli* O_111_	1 µg/ml	0.1	0.1
Heat-killed *S.* Enteritidis	10^7^ CE/ml	0.5	0.9
	10^8^ CE/ml	0.1	0.1
	10^9^ CE/ml	1.8	1.6
	10^10^ CE/ml	7.6	7.6
Viable *S.* Enteritidis	10^5^ CE/ml	0.0	1.2
	10^6^ CE/ml	0.0	0.1
	10^7^ CE/ml	0.1	0.0
	10^8^ CE/ml	101.1	9.6

## Discussion

Stem cell research advances progressed quickly in recent years. There is a worldwide interest to explore the developmental potential of stem cells in both basic and applied research questions. This requires a suitable model system that allows an extremely realistic study of stem cells, while ethically harmless.

In the present study, the cell line MuMac-E8 was analyzed in terms of their stem cell properties and their differentiation potential to investigate their suitability as a model system for differentiation. Initial experiments delivered clear indications of a stem-cell like phenotype of these cells. Therefore, we intended to investigate the gene expression profile of this cell line at the mRNA level. For this purpose, probe-based real-time RT-PCR assays were established for various stem-cell associated markers and for markers of differentiation.

The aim was a detailed description of the cells based on their marker profile at the mRNA level. This should enable a statement about their position within the stem cell hierarchy and the hematopoietic lineage. For this reason, a wide range of previously described markers was investigated in this work, including pluripotency and embryonic markers, markers for hematopoietic, mesenchymal and neural stem cells and markers for already differentiated cells.

Surprisingly, the performed gene expression analysis did not show a consistent pattern of expression for certain markers. This makes it difficult to draw conclusions from these findings for the differentiation state of MuMac-E8 cells. A possible explanation for this process is the inhomogeneity of the bulk cell culture. MuMac-E8 cells showed a divergent microscopic morphology. It might be a heterogenic cell population, which therefore may have different marker profiles.

MuMac-E8 cells were studied for the mRNA expression of several pluripotency markers. Nanog is a transcription factor that plays a crucial role in maintaining self-renewal of pluripotent embryonic stem cells [21], [22]. The expression of Nanog could be evidence of self-renewal potential of these cells. Nucleostemin (NST) was detected as another marker of pluripotency. This p53-binding protein is expressed both in embryonic stem cells and in stem cells of the central nervous system, but not in adult tissue cells [23]. NST is most likely also involved in the self-renewal of stem cells [24]. Both Nanog and NST, however, have been described in association with cancer [25]–[27]. The expression of the surface protein endoglin (CD105) has been frequently described in connection with mesenchymal stem cells (MSC) [28]–[30]. Like many other described markers for MSC, CD105 is also expressed on other cell types such as monocytes/macrophages [31] or early hematopoietic precursors [32].

The expression of hematopoietic markers such as CD45, CD14 and CD11b is strictly in contradiction to a MSC phenotype of MuMac-E8 cells. The absence of such markers is considered an accepted criterion for MSC [33], [34].

Sca-1, CD90.1 and CD117 presumed to be characteristic markers for murine hematopoietic stem cells (HSC) [35]–[38]. In the present study, MuMac-E8 cells were shown to be positive for Sca-1. There is evidence that Sca-1 plays a crucial role in the lineage-specific differentiation of hematopoietic stem cells and thus also in the development of progenitor cells [39], [40]. In conclusion from the results of the gene expression analysis for HSC it can be stated that the tested cell line believed to be hematopoietic cells at an advanced stage of differentiation. The expression of CD11b, F4/80, CD14, and CD64 suggests a myeloid phenotype. The occurrence of the marker EPCR delivered evidence for monocytes or macrophage-like cells. However, MuMac-E8 cells do also express the HSC marker Sca-1.

Finally, the study of neural markers revealed the expression of Ezrin and Pax-6 in MuMac-E8 cells. During the experiment, both markers showed a decrease in mRNA expression until day 30. Ezrin was demonstrated in the central nervous system of human, mouse and rat. Pax-6 is a transcription factor which is already expressed during the early embryogenesis and plays a crucial role in the development of the CNS and the eye [41]. Thus, at the mRNA level, MuMac-E8 cells show neuronal properties accordingly. In conclusion from these data it has to be stated that the mRNA expression profile does not allow a clear definition of the differentiation potential of MuMac-E8 cells. Thus, a definite classification of MuMac-E8 cells to belong to stem cells or hematopoietic cells was not possible from this approach. For this reason, *in-vitro* differentiation assays and functional testing of MuMac-E8 cells *in vivo* using lethally irradiated mice were performed.

MuMac-E8 cells showed a marked colony formation in the CFU assay. These colonies were composed of numerous cells which showed a very uniform round shape. In comparison with recordings of various colony types these cells showed similarity with myeloid colony forms. Scarcely cell growth was observed in the periphery of the colonies. Accordingly, it is probably the colony type CFU-M. MuMac-E8 cells showed a clonal growth but they are obviously not able to differentiate into various specialized cell types of the hematopoietic lineage.

On the basis of *in-vivo* experiments in lethally irradiated mice, it was studied whether MuMac-E8 cells are able to reconstitute the hematopoietic system in mice. The analysis of the blood cell profile in terms of existing white blood cell count showed a strong decrease of leukocytes during the course of the experiment. Accordingly, MuMac-E8 cells in applied cell numbers were not able to reconstitute the hematopoietic system in mice. However, it can be assumed that this is not a methodological error. At the same time the repopulation of hematopoietic cells is possible by transplantation of bone marrow and bone-marrow derived hematopoietic stem cells [13]–[17]. Only at the mRNA level these cells revealed some stem cell properties, but they did not show any *in-vivo* differentiation potential to hematopoietic cells. Hence, this data let conclude, that the cell line characterized in this work represents a new monocyte/macrophage or monocyte/macrophage precursor cell line rather than a stem cell line, which is less adherent than other cell lines derived from macrophages. For the first time, these cells could be clearly characterized by real-time RT-PCR and colony-forming cell assays. Transplantation into lethally irradiated triple-transgenic mice revealed no reconstruction of the hematopoietic system indicating that this cell line has no pluripotent capacity, suggesting also a differentiated myeloid rather than a stem cell phenotype of this cell line.

MuMac-E8 cells were firstly isolated from joint tumors in human RA-fibroblast induced human/murine SCID arthritis [1]. A gene-therapeutic approach using interleukin(IL)-4- or IL-10-transfected NIH3T3 cells did not decrease the joint inflammation as expected but in contrast caused an joint-destroying tumor. From these tumors several cell lines were recruited and subsequently the MuMac-E8 cell line was subcloned from one of the primary tumor cell lines. The cells are of murine origin and at least 3 passages were generated. After isolation, the clonality of MuMac-E8 was not investigated by a molecular method. The cell line was cloned by limiting dilution (3 times) to one single cell. From this single cell, a cell population with heterogeneous characteristics and regenerative potential was cultured. Before determination of selected genes, MuMac-E8 cells were synchronized because of their heterogeneity in bulk culture to enable a uniform start of the cells and to exclude the influence of genes responsible for the cell cycle. In further experiments, the protein expression profiles related to the gene expression should be analyzed also in comparison to embryonic stem cells (i.e. Nanog) or neural cells (i.e. Pax-6). However, the determination of the corresponding protein will not prove its functionality. Therefore, further functional assays should be performed. MuMac-E8 cells also showed expression of Oct4 (unpublished data from qualitative endpoint RT-PCR). To determine the pluripotency of MuMac-E8, also Sox2 and the potential of tri-lineage teratoma formation should be analyzed in more detail. Sca-1 (Ly6F) is also highly expressed on the surface of macrophages and more moderately expressed on B- and T-lymphocytes and in combination with c-kit on hematopoietic stem cells. Also, hematopoietic cells could be identified *in vitro* using May-Grünwald-Giemsa staining. The stained cells are easily to identify due to their typical morphology. The expression of c-kit proves that there is a subpopulation with hematopoietic stem cell properties within the entire MuMac-E8 cell population. Because MuMac-E8 cells could be easily cultured in RPMI 1640 medium supplemented with FCS, it would be interesting to compare the expression profiles and functionality with MuMac-E8 cells cultured in other media (e. g. DMEM) because RPMI 1640 is not the standard medium to culture stem cells.

As shown in this work, the cell line MuMac-E8 is suitable as an *in-vitro* model for testing of macrophage functions (e.g. immunotoxicity or immunopharmacological testing). The cell line MuMac-E8 is easy to manufacture and modest regarding the cell culture process. This cell line very likely consists of myeloid precursors that showed characteristics of macrophages.

It can be concluded that the MuMac-E8 cell line has the potential to differentiate into macrophages based on the immunophenotype, the phagocytic potential and NO producing capacity of the MuMac-E8 cells. Macrophages, dendritic cells and granulocytes can arise from a common myeloid precursor through different stimulation protocols [42]. However, neither GM-CSF nor IL-4 was added to the cell culture of MuMac-E8 cells. Because myeloid cells can show a high plasticity among which macrophages [43]–[50], it should be investigated whether addition of different stimuli, e.g. IL-4, GM-CSF, M-CSF or tumor-conditioned medium [6] to the MuMac-E8 cell line gives rise to distinct cell types.

Therefore, different culture conditions should be prepared and the phenotype of the arising cells with focus on markers that are able to discriminate between type 1 versus type 2 macrophages [51], dendritic cells [52], monocytic [53] versus granulocytic myeloid derived suppressor cells [54] should be determined. If distinct cell populations are obtained, it would be worthwhile to compare the functionality of these cells after stimulation and with regard to the inhibition of T cells. Hence, MuMac-E8 cells could be a potential tool to study these differentiated cell populations. Especially myeloid suppressor cells that are found in the microenvironment of cancers [6] could be studied regarding their influences on the tumor growth [6], [8] or the immune cell activation. In this view, the cell line might also be a valuable tool to study such issues without the need of additional animal experiments.

Despite MuMac-E8 cells represent maturated cells, typical pluripotency markers (e. g. Nanog, Nucleostemin) could still be determined on the transcript level but appeared to be silenced. It has to be proven, whether MuMac-E8 cells can be used for reprogramming to cells with stem cell characteristics, which will be investigated in future projects as well.

Phenotypically MuMac-E8 cells revealed a macrophage-like morphology and behaviour as shown by fluorescence staining of F-actin or impedance-based RTCA. Phagocytosis of heat-killed salmonellae was accompanied by increased surface expression of CD14 and CD64 (FcγRI). These molecules are involved in recognition and receptor-mediated phagocytosis of bacteria, respectively. Thus, MuMac-E8 cells show important features of mature macrophages. Moreover, the induction of iNOS-dependent NO synthesis by living salmonellae supports the macrophage phenotype of MuMac-E8 cells.

## Conclusions

Although, it could be shown that MuMac-E8 cells express some genes associated with pluripotent stem cells (i.e. Nanog, Nucleostemin) as well as genes for hematopoietic markers (i.e. EPCR, Sca-1, CD11b, CD45), for the mesenchymal marker CD105, and for the neural markers Pax-6 and Ezrin at the mRNA level, these cells exhibited no stem-cell like phenotype. However, the expression of important myeloid markers (i.e. CD11b, F4/80, CD14, CD64) at the protein level together with the adherence behavior and the morphology of MuMac-E8 cells in culture as demonstrated by the RTCA result or the May-Grünwald-Giemsa and the Phalloidin-Alexa staining of MuMac-E8 cells, respectively, revealed a macrophage-like phenotype. This data let conclude, that the MuMac-E8 cell line is a new monocyte/macrophage or late-stage monocyte/macrophage precursor cell line which is less adherent than other well-described murine macrophage cell lines such as RAW 264.7.

## Supporting Information

S1 Video **Time-lapse video of MuMac-E8 cells in culture.** Non-adherent and adherent cells could be observed side by side. While the non-adherent cells seemed to represent cells in the mitotic phase, adherent cells seemed to represent either a post-mitotic phenotype in the G_0_ phase or the inter-phase phenotype (G_1_, S- or G_2_-phase) of the cell cycle, as concluded from observations in several time-lapse videos of MuMac-E8 cells cultured under conventional conditions.
(MPEG)
