# Correction: Characterization of the Murine Myeloid Precursor Cell Line MuMac-E8

**DOI:** 10.1371/journal.pone.0119657

**Published:** 2015-04-13

**Authors:** The PLOS ONE Staff

There is an error in the “Ethics statement” section of this article. The correct statement is: All mice were housed, treated and handled under permission and in accordance with the guidelines of the Animal Care Committee of the University of Leipzig and the Regional Board of Animal Care for the district of Leipzig (Landesdirektion Sachsen, Leipzig, approval number TVV 55/11). The Animal Care Committee of the University of Leipzig and the Regional Board of Animal Care for the district of Leipzig specifically approved this study.
